# Whole-genome sequencing of 490,640 UK Biobank participants

**DOI:** 10.1038/s41586-025-09272-9

**Published:** 2025-08-06

**Authors:** The UK Biobank Whole-Genome Sequencing Consortium, Keren Carss, Bjarni V. Halldorsson, Liping Hou, Jimmy Liu, Eleanor Wheeler, Yancy Lo, Kousik Kundu, Zhuoyi Huang, Ben Lacey, Ryan S. Dhindsa, Diana Rajan, Jelena Randjelovic, Neil Marriott, Carol E. Scott, Ahmet Sinan Yavuz, Ian Johnston, Trevor Howe, Mary Helen Black, Kari Stefansson, Robert Scott, Slavé Petrovski, Shuwei Li, Adrian Cortes, Keren Carss, Eleanor Wheeler, Kousik Kundu, Ryan S. Dhindsa, Slavé Petrovski, Fengyuan Hu, Quanli Wang, Oliver S. Burren, Sri V. V. Deevi, Carolina Haefliger, Kieren Lythgow, Peter H. Maccallum, Karyn Mégy, Jonathan Mitchell, Sean O’Dell, Amanda O’Neill, Katherine R. Smith, Haeyam Taiy, Menelas Pangalos, Ruth March, Sebastian Wasilewski, Bjarni V. Halldorsson, Kari Stefansson, Hannes P. Eggertsson, Kristjan H. S. Moore, Hannes Hauswedell, Ogmundur Eiriksson, Aron Skaftason, Nokkvi Gislason, Svanhvit Sigurjonsdottir, Magnus O. Ulfarsson, Gunnar Palsson, Marteinn T. Hardarson, Asmundur Oddsson, Brynjar O. Jensson, Snaedis Kristmundsdottir, Brynja D. Sigurpalsdottir, Olafur A. Stefansson, Doruk Beyter, Guillaume Holley, Vinicius Tragante, Arnaldur Gylfason, Pall I. Olason, Florian Zink, Margret Asgeirsdottir, Sverrir T. Sverrisson, Brynjar Sigurdsson, Sigurjon A. Gudjonsson, Gunnar T. Sigurdsson, Gisli H. Halldorsson, Gardar Sveinbjornsson, Unnur Styrkarsdottir, Droplaug N. Magnusdottir, Steinunn Snorradottir, Kari Kristinsson, Emilia Sobech, Gudmar Thorleifsson, Frosti Jonsson, Pall Melsted, Ingileif Jonsdottir, Thorunn Rafnar, Hilma Holm, Hreinn Stefansson, Jona Saemundsdottir, Daniel F. Gudbjartsson, Olafur T. Magnusson, Gisli Masson, Unnur Thorsteinsdottir, Agnar Helgason, Hakon Jonsson, Patrick Sulem, Jimmy Liu, Yancy Lo, Robert Scott, Adrian Cortes, Jatin Sandhuria, Tom G. Richardson, Laurence Howe, Chloe Robins, Dongjing Liu, Patrick Albers, Mariana Pereira, Daniel Seaton, Yury Aulchenko, John Whittaker, Manolis Dermitzakis, Toby Johnson, Jonathan Davitte, Erik Ingelsson, Liping Hou, Trevor Howe, Mary Helen Black, Shuwei Li, Julio Molineros, Yanfei Zhang, Alexander H. Li, Evan H. Baugh, Elisabeth Mlynarski, Abolfazl Doostparast Torshizi, Gamal Abdel-Azim, Brian Mautz, Karen Y. He, Jingyue Xi, Shirley Nieves-Rodriguez, Asif Khan, Songjun Xu, Xingjun Liu, Brice Sarver, Dongnhu Truong, Mohamed-Ramzi Temanni, Christopher D. Whelan, Letizia Goretti, Najat Khan, Belen Fraile, Tommaso Mansi, Guna Rajagopal, Diana Rajan, Jelena Randjelovic, Neil Marriott, Carol E. Scott, Ahmet Sinan Yavuz, Ian Johnston, Shaheen Akhtar, Siobhan Austin-Guest, Robert Barber, Daniel Barrett, Tristram Bellerby, Adrian Clarke, Richard Clark, Maria Coppola, Linda Cornwell, Abby Crackett, Joseph Dawson, Callum Day, Alexander Dove, Jillian Durham, Robert Fairweather, Marcella Ferrero, Michael Fenton, Howerd Fordham, Audrey Fraser, Paul Heath, Emily Heron, Gary Hornett, Lena Hughes-Hallett, David K. Jackson, Alexander Jakubowski Smith, Adam Laverack, Katharine Law, Steven R. Leonard, Kevin Lewis, Jennifer Liddle, Alice Lindsell, Sally Linsdell, Jamie Lovell, James Mack, Henry Mallalieu, Irfaan Mamun, Ana Monteiro, Leanne Morrow, Barbora Pardubska, Alexandru Popov, Lisa Sloper, Jan Squares, Ian Still, Oprah Taylor, Sam Taylor, Jaime M. Tovar Corona, Elliott Trigg, Valerie Vancollie, Paul Voak, Danni Weldon, Alan Wells, Eloise Wells, Mia Williams, Sean Wright, Nevena Miletic, Lea Lenhardt Ackovic, Marijeta Slavkovic-Ilic, Mladen Lazarevic, Louise Aigrain, Nicholas Redshaw, Michael Quail, Lesley Shirley, Scott Thurston, Peter Ellis, Laura Grout, Natalie Smerdon, Emma Gray, Richard Rance, Cordelia Langford, Ben Lacey, Rory Collins, Mark Effingham, Naomi Allen, Jonathan Sellors, Simon Sheard, Mahesh Pancholi, Caroline Clark, Lucy Burkitt-Gray, Samantha Welsh, Daniel Fry, Rachel Watson, Lauren Carson, Alan Young, Zhuoyi Huang, Rami Mehio, Ole Schulz-Trieglaff

**Affiliations:** 1 Centre for Genomics Research, Discovery Sciences, BioPharmaceuticals R&D, AstraZeneca, Cambridge, UK; 2 Amgen deCODE genetics, Reykjavik, Iceland; 3 School of Technology, Reykjavik University, Reykjavik, Iceland; 4 AI/ML, Data Science & Digital Health, Janssen Research & Development, Spring House, PA USA; 5 Human Genetics & Genomics, GSK, Collegeville, PA USA; 6 Population Genetics, Illumina, San Diego, CA USA; 7 UK Biobank, Stockport, UK; 8 Centre for Genomics Research, Discovery Sciences, BioPharmaceuticals R&D, AstraZeneca, Waltham, MA USA; 9 Wellcome Sanger Institue, Hinxton, UK; 10 Velsera, Charlestown, MA USA; 11 External Innovation, Data Science & Digital Health, Janssen Research & Development, London, UK; 12 Faculty of Medicine, School of Health Sciences, University of Iceland, Reykjavik, Iceland; 13 Human Genetics & Genomics, GSK, Stevenage, UK; 14 Human Genetics & Genomics, GSK, Heidelberg, Germany; 15 ELIXIR, Hinxton, UK; 16 BioPharmaceuticals R&D, AstraZeneca, Cambridge, UK; 17 Precision Medicine & Biosamples, Oncology R&D, AstraZeneca, Cambridge, UK; 18 School of Engineering and Natural Sciences, University of Iceland, Reykjavik, Iceland; 19 Department of Anthropology, University of Iceland, Reykjavik, Iceland; 20 R&D Data Science & Data Engineering, Collegeville, PA USA; 21 DS NS, Data Science & Digital Health, Janssen Research & Development, Boston, MA USA; 22 External Innovation, Discovery, Product Development & Supply, Janssen Research & Development, Beerse, Belgium; 23 Data Science & Digital Health, Janssen Research & Development, Spring House, PA USA; 24 Computational Science, Discovery, Product Development & Supply, Janssen Research & Development, Spring House, PA USA; 25 Software Informatics, Illumina, San Diego, CA USA; 26 Population Genetics, Illumina, Cambridge, UK; 27 Present Address: Foresite Labs, Boston, MA USA; 28 Present Address: MRC Biostatistics Unit, University of Cambridge, Cambridge, UK; 29 Present Address: ZS Discovery, Evanston, IL USA; 30 Present Address: Alia Therapeutics SRL, Milano, Italy; 31 Present Address: Recursion, Salt Lake City, UT USA; 32 Present Address: Samsara BioCapital, Palo Alto, CA USA

**Keywords:** Genetics research, Next-generation sequencing, Rare variants, Genome-wide association studies

## Abstract

Whole-genome sequencing provides an unbiased and complete view of the human genome and enables the discovery of genetic variation without the technical limitations of other genotyping technologies. Here we report on whole-genome sequencing of 490,640 UK Biobank participants, building on previous genotyping effort^1^. This advance deepens our understanding of how genetics associates with disease biology and further enhances the value of this open resource for the study of human biology and health. Coupling this dataset with rich phenotypic data, we surveyed within- and cross-ancestry genomic associations and identified novel genetic and clinical insights. Although most associations with disease traits were primarily observed in individuals of European ancestries, strong or novel signals were also identified in individuals of African and Asian ancestries. With the improved ability to accurately genotype structural variants and exonic variation in both coding and UTR sequences, we strengthened and revealed novel insights relative to whole-exome sequencing^2,3^ analyses. This dataset, representing a large collection of whole-genome sequencing data that is available to the UK Biobank research community, will enable advances of our understanding of the human genome, facilitate the discovery of diagnostics and therapeutics with higher efficacy and improved safety profile, and enable precision medicine strategies with the potential to improve global health.

## Abstract (web summary)

A study reports whole-genome sequences for 490,640 participants from the UK Biobank and combines these data with phenotypic data to provide new insights into the relationship between human variation and sequence variation.

## Main

The UK Biobank (UKB) is a population‐based study that collected detailed information from 490,640 UK participants, including biological samples and comprehensive health‐related and demographic measures^1^. Numerous subsequent data collection and generation efforts, including multimodal brain imaging^4^, proteomics^5^, metabolomics^6^ and others, have markedly increased the depth of the dataset. Here we present a step change in the UKB resource, and for the life sciences, with the completion of whole-genome sequencing (WGS) in 490,640 participants. In the original release, all samples were genotyped^1^ and imputed to about 96 million single nucleotide polymorphisms (SNPs). SNP genotyping and imputation allow the accurate characterization of relatively common variants, but these technologies are not suitable for rare genetic variation and complex regions of the genome. UKB samples also underwent whole-exome sequencing^7^ (WES), which allows for characterization of the 2–3% of the genome that is exonic but omits nearly all non-coding variation and is limited in the detection of structural variants (SVs). Rare non-coding variation is known to contribute to human diseases and other complex traits, although this remains relatively understudied^8–10^. This large-scale, deeply phenotyped WGS dataset brings enormous potential to expand our understanding of the role of rare non-coding variation in health and disease.

We demonstrate the utility of WGS in the identification of about 1.5 billion variants (comprising SNPs, insertion–deletion (indel) variants and SVs) in the UKB participants. We observed an 18.8-fold and greater than 40-fold increase in observed human variation compared to imputed array and WES, respectively. These variants were associated with many disease features and traits, enabling improved characterization of disease mechanisms, such as variants influencing disease risk through non-coding mechanisms. These data can be used to address multiple drug discovery and development questions, including target selection, validation, assessment of safety concerns, identification of patient populations with specific underlying genetic drivers of disease, and repositioning opportunities^11,12^. A valuable unique benefit is that these data will facilitate an improved understanding of the selective constraints acting on disruption outside the coding genome, which will improve the ability to prioritize rare non-coding variants with a large effect on disease risk^13^.

**Extended Data Fig. 1:**
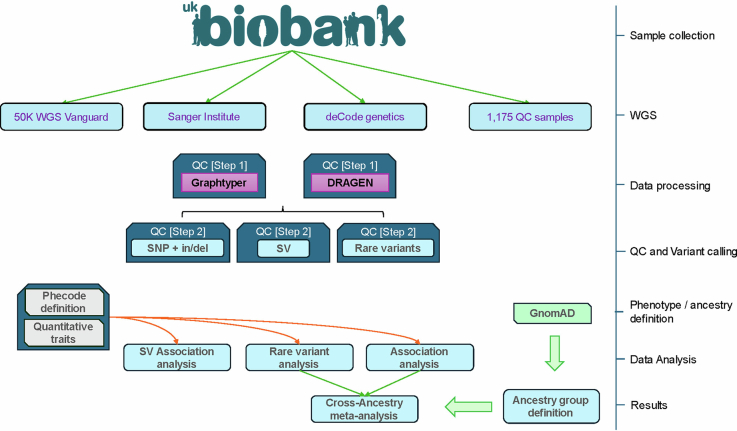
Graphical summary: framework followed in this UK Biobank study. Participant’s sample were collected from UK Biobank and underwent whole genome sequencing as described in the Supplementary Information. Sequencing data was analyzed with two distinct bioinformatic pipelines generating datasets GraphTyper and DRAGEN, both datasets, and the followed by variant calling of SNPs, indels, and structural variants (SV). Participants were identified to one of five ancestry groups for association analysis of genetic variants for a series of disease endpoints and quantitative traits. Cross-ancestry meta-analysis was then performed. The UK Biobank logo is reproduced with permission.

This resource will enable exploration of human genetic variation and its effect on disease pathogenesis. The aims of the current study are twofold: to describe and characterize the UKB 490,640 WGS resource; and to highlight some initial examples of unique insights and future avenues for exploration (summarized in Extended Data Fig. 1).

## Data processing

### Sequencing

The whole genomes of 490,640 UKB participants were sequenced to an average coverage of 32.5× (with at least 23.5× per individual; Supplementary Fig. 1) using Illumina NovaSeq 6000 sequencing machines; in addition, 1,175 samples were sequenced in duplicate for quality control purposes (Supplementary Methods).

### Cohorts

We defined five cohorts with distinct ancestry in the UKB WGS dataset using a classifier trained with data from the Genome Aggregation Database^14^ (gnomAD; Supplementary Methods), which identified 9,229 participants being of African ancestry (AFR), 2,869 of Ashkenazi Jewish ancestry (ASJ), 2,245 of East Asian ancestry (EAS), 458,855 of non-Finnish European ancestry (NFE) and 9,674 of South Asian ancestry (SAS), and the remaining 7,768 individuals of other ancestries or non-confidently assigned to one group. Most individuals (93.5%) were of non-Finnish European ancestry, with the remaining 31,785 individuals representing other continental populations. Although this resource is largely European, this effort also marks an extensive WGS effort so far in non-European individuals (Supplementary Fig. 2). The increase is notable in the SAS group, where the UKB WGS SAS cohort is two times larger than any other WGS cohort of this ancestry available in gnomAD v3^15,16^ (2,419 SAS individuals), the 1000 Genomes Project^17^ (601), Trans-Omics for Precision Medicine^18^ (4,599)^18^ or the Human Genome Diversity Project^19^ (181).

### SNPs and indels

This study reports findings from three different SNP and indel datasets: joint calling across all individuals using GraphTyper; single-sample calling with DRAGEN 3.7.8; and multi-sample aggregated DRAGEN 3.7.8 dataset release 2 (Supplementary Methods). This diversity of approaches reflects developments of these methods throughout the course of this project and gives the opportunity to explore the various workflows used by consortium members and other users of the UKB.

**Fig. 1:**
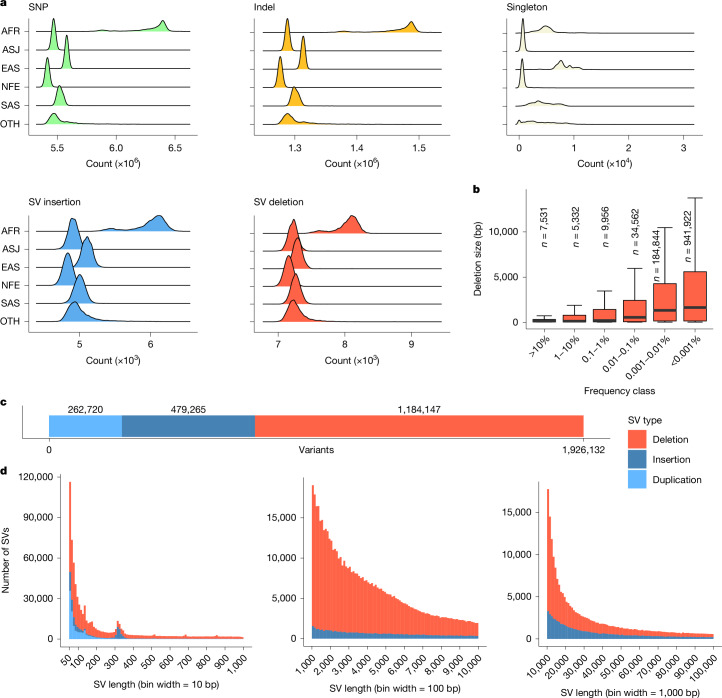
Variant call sets. **a**, The density (counts) of the per-individual number of variants split up by the five populations considered in this study from the GraphTyper call sets. Panels show number of SNPs, indels, singleton SNPs and indels, combined number of SV insertions and duplications and SV deletions. **b**, The length of SV deletions discovered in this study, split by the frequency of the variant. Data are represented as box plots; the middle line represents the median, the lower and upper part of the red box plot correspond to the first and third quartiles, and the upper whisker extends from the 75th percentile to the 95th percentile. *n* indicates the number of SV deletions per frequency bin. **c**, The number of variants discovered split by variant class (duplication, insertion and deletion). **d**, The size of insertions and deletions discovered shown in range from 50 bp up to 1,000 bp, 10,000 bp and 100,000 bp.

We called 1,037,556,156 SNPs and 101,188,713 indels using GraphTyper (Fig. 1a). Most variants, 1,025,188,151 (98.80%) SNPs and 97,190,353 (96.05%) indels, were reliable (AAscore >0.5 and <5 duplicate inconsistencies; Supplementary Methods). All GraphTyper analyses are restricted to this set unless otherwise noted. The number of variants identified in at least 1 individual using GraphTyper was 42 times larger than the number of variants identified through WES^7^ (Table 1 and Supplementary Methods). Notably, in the WES dataset, variants in exons that are transcribed but not translated were missed; 69.2% and 89.9% of the 5′ and 3′ untranslated region (UTR) variants are missing from the WES dataset, respectively. We estimate that, even inside coding exons curated by ENCODE^20^ at present, 13.7% of variants are missed in the WES dataset (Table 1 and Supplementary Tables 1 and 2). A subset of the missed variants is explained by the 25,853 fewer samples that are available in the WES dataset release. Manual inspection of a subset of the missing variants in the WES dataset, in which both whole-exome and whole-genome calls were available, suggests that these are absent owing to both missing coverage in some regions and genotyping filters. Almost all variants identified in the WES dataset are found in the WGS dataset (Table 1).

**Table 1:** Numbers of variants identified in at least one individual stratified by annotation across the GraphTyper dataset, using Ensembl version 101 annotations comparing WES and WGS data releases

Annotation	WGS	WES	Intersection	Union	Unique to WES	Present WES (%)	Missing WES (%)	Present WGS (%)	Missing WGS (%)
**Coding**	12,563,849	10,997,033	10,813,189	12,747,693	183,844	86.267	13.733	98.558	1.442
**Splice**	922,111	799,114	784,865	936,360	14,249	85.343	14.657	98.478	1.522
**5′ UTR**	3,127,742	973,615	944,458	3,156,899	29,157	30.841	69.159	99.076	0.924
**3′ UTR**	13,941,989	1,406,375	1,366,180	13,982,184	40,195	10.058	89.942	99.713	0.287
**Proximal**	490,613,217	12,482,022	11,988,515	491,106,724	493,507	2.542	97.458	99.900	0.100
**Intergenic**	601,209,600	182,763	165,217	601,227,146	17,546	0.030	99.970	99.997	0.003
**Sum**	1,122,378,508	26,840,922	26,062,424	1,123,157,006	778,498	2.390	97.610	99.931	0.069

Percentages are based on the number of variants compared to the union across WES and WGS variants per annotation type. Supplementary Tables 1 and 2 show stratified versions of this table.

We compared the DRAGEN single-sample WGS dataset to the previously published DRAGEN WES dataset^21^ to explore the number of variants identified across coding, splice and 5′ and 3′ UTR annotation categories. As previously described^22^, a greater number of variants were captured in the WGS data across all annotation categories, with most (98.26%–99.67%) variants identified in the WES dataset being captured in the WGS data (Table 2). WES did not capture many of the UTR variants, particularly 3′ UTR variants, for which only 24.78% of variants present across both datasets were found in the WES data, compared to 99.67% in the WGS data (Table 2). Notably, the pattern of variant numbers was generally similar between GraphTyper and DRAGEN single-sample datasets.

**Table 2:** Numbers of variants identified in at least one individual stratified by annotation across the DRAGEN single-sample dataset annotated using SnpEff v4.3 against Ensembl Build 38.92

Annotation	WGS	WES	Intersection	Union	Unique to WES	Present WES (%)	Unique to WGS	Present WGS (%)
**Coding**	12,226,571	11,596,546	11,522,471	12,300,646	74,075	94.28%	704,100	99.40%
**Splice**	1,180,346	1,107,034	1,086,157	1,201,223	20,877	92.16%	94,189	98.26%
**5**′ **UTR**	4,867,014	1,892,335	1,859,132	4,900,217	33,203	38.62%	3,007,882	99.32%
**3**′ **UTR**	16,211,884	4,030,034	3,976,725	16,265,193	53,309	24.78%	12,235,159	99.67%

For DRAGEN, high-quality variant counts are limited to the 460,552 samples for which we had both WES and WGS available. Percentages are based on the number of variants compared to the union across WES and WGS variants per annotation type. Supplementary Table 3 shows a stratified version of this table.

Using the DRAGEN aggregated dataset release 2, we called 1,081,661,407 PASS SNPs and 129,273,976 PASS indels on autosomes, sex chromosomes, mitochondria and alternative contigs of the whole cohort (Supplementary Table 3).

Quality assessment is based on Genome in a Bottle samples extracted from the joint call set after cohort-level filtering (genotype inconsistency among 1,043 trios and between 177 monozygotic twins) and cohort-level genotype missingness. In high-confidence regions, for Genome in a Bottle samples, the sensitivity and precision of PASS SNPs are 98.95% and 99.97%, respectively, and the sensitivity and precision of PASS indels are 97.43% and 99.85%, respectively (Supplementary Table 4). In autosomes, for trios, genotype inconsistency of PASS variants is 0.029% in high-confidence regions, and 0.829% in low-confidence regions. For twins, genotype inconsistency of PASS variants is 0.036% in high-confidence regions, and 1.650% in low-confidence regions (Supplementary Table 5). Across the cohort, genotype missingness of PASS variants is 0.005% in high-confidence regions, and 0.010% in low-confidence regions (Supplementary Table 6).

**Extended Data Fig. 2:**
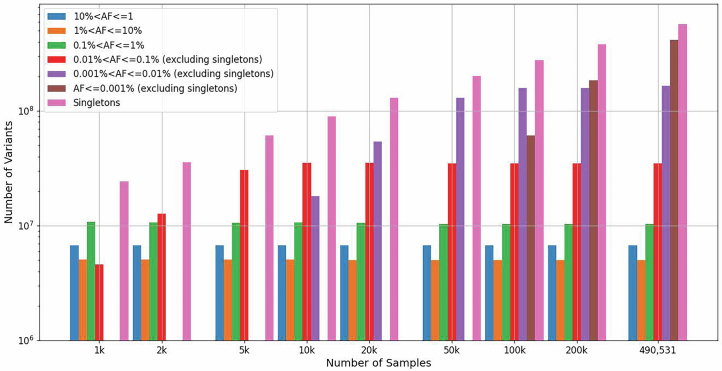
Effect of sample size on variant number. Number of variants in UK Biobank DRAGEN aggregated variant dataset (release 2 PASS variants) in different allele frequency ranges as the number of samples increase from 1000 to 490,541 (based on random downsampling). Variant alleles are collected from all autosomes, sex chromosomes, mitochondria, and ALT contigs.

Using random downsampling of samples, we investigated the gain in number of variants in the UKB DRAGEN aggregated variant dataset as sample size increases from 1,000 to 490,541 (Extended Data Fig. 2). As expected, for common variants (for example, >1% frequency), we do not observe an increase in number of variants with increasing sample size, but for the rarest variants (for example, ≤0.001% frequency), we observe substantial increases in number of variants with sample size, even at the highest sample size, supporting the value of continuing very large-scale sequencing efforts to discover novel and high-impact rare variants (Extended Data Fig. 2).

### SVs

We identified SVs in each individual using the DRAGEN SV caller and combined these with variants from a long-read study^23^ and the assemblies of seven individuals^24^. The resulting 2,739,152 SVs were genotyped with GraphTyper^24^, of which 70.3% (1,926,132; Fig. 1b) were considered reliable (Supplementary Methods); 262,720 duplications, 479,265 insertions and 1,184,147 deletions. SVs were defined as variants being at least 50 base pairs (bp) and the size distribution showed a well-documented skew towards short variants (Fig. 1d).

On average, we identified 13,102 reliably called SVs per individual; 7,340 deletions and 5,762 insertions or duplications (Fig. 1b). These numbers are greater than the 7,439 SVs per individual found by gnomAD-SV^25^, another short-read study, but considerably smaller than the 22,636 high-quality SVs found in a long-read sequencing study^23^, mostly owing to an under-representation of insertions and SVs in repetitive regions. Despite the number of SVs being much smaller than the number of SNPs and indels, the number of base pairs affected per haploid genome on average (3.6 Mb) is comparable to that of SNPs (2.9 Mb) and indels (1.5 Mb). Most of the SVs are very rare; 1,470,329 (76.3%) are carried by fewer than 10 individuals (<0.001% frequency). We observed that rare variants are generally longer than common variants with a median length of 1,660 bp for deletions carried by fewer than 10 individuals and 169 bp for deletions with frequency above 1% (Fig. 1b).

Variant identification was performed analogously to that for the UKB 150,119 release^22^ but replacing Manta^26^ with the DRAGEN SV caller, which identifies a greater number of insertions. Owing to the improved discovery step and a modified variant filtering procedure, the number of reliably called SVs is approximately threefold larger in the current set compared to the previous release^22^. Out of the 637,321 SVs reliably called in our previous call set, 590,037 (92.6%) are also reliably called in the current call set. An additional 11,958 (1.8%) were part of the genotyping set but no longer considered reliable when genotyped, and the remaining 35,327 (5.5%) were not part of the current set of variants.

The number of variants called per individual varies by population, with the largest number of variants called in individuals in the AFR cohort, followed by the EAS, SAS, ASJ and finally the NFE cohort, for which individuals had the lowest number of called variants when compared to the current reference genome (Fig. 1a).

## Phenotype associations

We integrated deep phenotyping data^27^ available for most UKB participants and performed genetic association analysis across selected disease outcomes captured with electronic health records and molecular and physical measurement phenotypes, many of which are well-established disease biomarkers. Association testing was performed for all observed genetic variants and using several genetic models; we included single-variant tests, multi-ancestry meta-analysis, rare-variant collapsing analysis and SV analysis (Supplementary Methods).

### Genome-wide association analysis

Genome-wide association analysis for individual SNPs and small indels was performed using the GraphTyper dataset in each ancestry cohort for 764 ICD-10 codes (*n* cases >200) and 71 selected quantitative phenotypes (*n* > 1,000; Supplementary Table 7). For the NFE cohort, we estimated the gain in discovery and improvement of fine mapping in association signals observed with the WGS call set versus variants observed in the imputed array genetic dataset^1^ using equivalent analysis results with the same cohort and phenotyping strategy. We observed that whereas the increase in discovery was modest for common variant associations (Supplementary Fig. 3), the ability to fine map association signals was improved, and this was not due only to the loss of power in association tests attributable to imputation accuracy in the array dataset. We identified 33,123 associations (*P* value < 5 × 10^−8^) across 763 binary and 71 quantitative genome-wide association study (GWAS) datasets (Supplementary Methods). Of these, 3,991 (12.05%) are new to the WGS data when compared to those identified using only array imputed variants. As expected, most associated variants novel to WGS are rare variants, including 86% of associations with minor allele frequency (MAF) <0.0001, whereas only 2% of associations with MAF > 0.1 are novel to WGS (Supplementary Fig. 3). Among the 29,357 associations identified using array imputed variants, 2,984 had a different, more significant, lead variant in the WGS variants, resulting in improved fine mapping of the association signals observed (Supplementary Table 8). For example, a common variant association uncovered by WGS that was previously missed by the imputed array data is near genes *MRC1* and *TMEM236* in chromosome 10, where we identified an association between rs371858405 (NFE MAF = 0.24) and reduced hypothyroidism risk (odds ratio (OR) = 0.94, *P* value = 2.6 × 10^−11^). In the imputed data, the region within the WGS lead variant has sparse SNP coverage when compared to adjacent regions (Supplementary Fig. 4b), probably a result of a patch to the hg19 reference genome (chr10_gl383543_fix) that occurred after the UKB genotyping array was designed. A second example illustrating a new biological findings with rare genetic variation is the observation of a rare frameshift variant (MAF = 5.1 × 10^−5^) in *FOXE3* chr. 1: 47417015:GC:G (rs1176723126) found to be significantly associated with the first occurrence phenotype ‘other cataract’ (ICD-10 code H26; *P* value = 6.2 × 10^−9^; Supplementary Fig. 4b). The link between *FOXE3* and cataract, and other ocular diseases, was reported in previous familial studies and human and mouse disease models^28^, but the association was not observed in the UKB imputed array or meta-analyses that included the UKB imputed array^29^.

### Multi-ancestry meta-GWAS

**Fig. 2:**
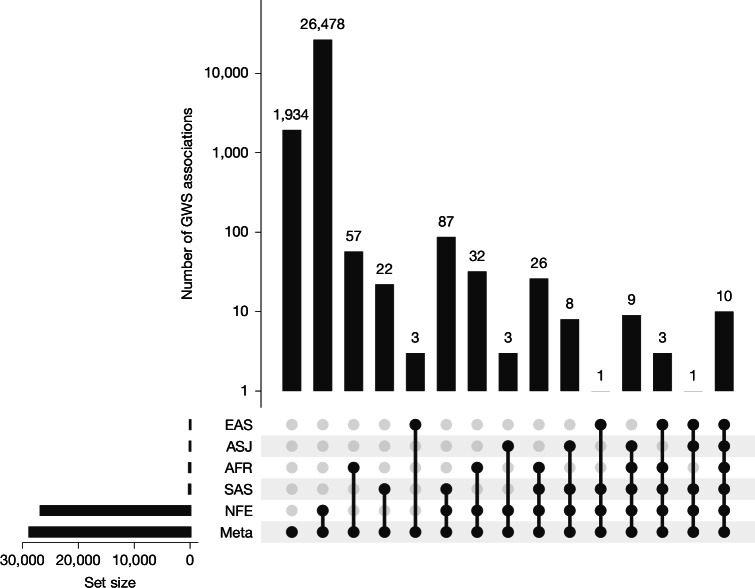
UpSet plot of GWS associations across ancestries. Ancestry labels are sorted by number of GWS associations in each set: meta-analysis (Meta), NFE, SAS, AFR, ASJ and EAS.

To examine multi-ancestry genetics of tested health-related phenotypes, we performed trans-ancestry meta-analysis of the GraphTyper GWAS data across 5 ancestries for 68 quantitative traits with ≥1,000 measurements in at least 2 ancestries and 228 ICD-10 disease outcomes with ≥200 cases in at least 2 ancestries. We identified 28,674 genome-wide significant (GWS; *P* value < 5.0 × 10^−8^) associations in the meta-analysis (Supplementary Methods, Supplementary Fig. 5 and Supplementary Table 9); of these, 1,934 associations were observed only in the meta-analysis, 26,478 were also observed in the NFE analysis, 82 were observed only in 1 of the non-NFE cohort analyses, and the remaining 180 associations were observed in more than 1 ancestry cohort (Fig. 2 and Supplementary Table 10). Among the 28,674 identified associations, 4,760 (16.6%) were not previously reported in the GWAS Catalog or OpenTargets^30^ (Supplementary Methods, Supplementary Fig. 3b and Supplementary Table 9).

**Extended Data Fig. 3:**
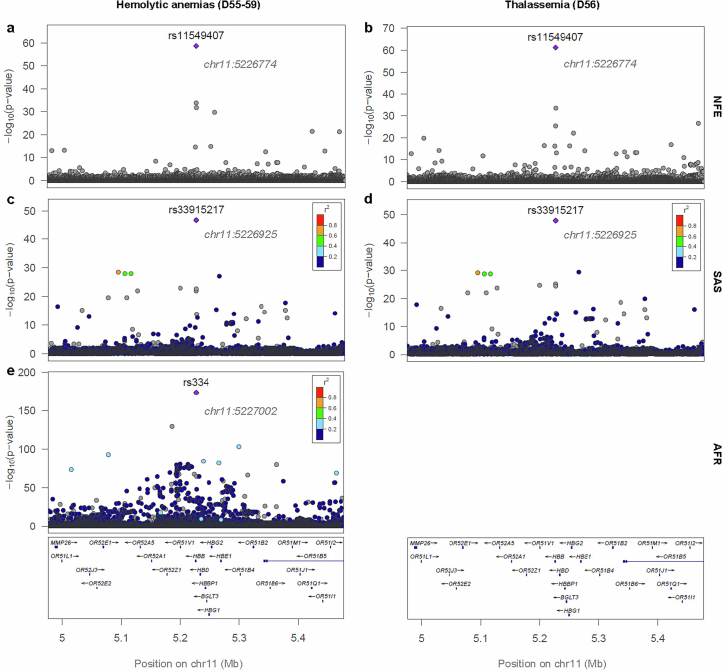
Regional plot for HBB-HBE1 locus associated with Hemolytic anemias (ICD10: D55-59) and Thalassaemia (ICD10: D56) in NFE, AFR, SAS populations. NFE: non-Finnish European; AFR: African; SAS: South Asian; EAS: East Asian. GWAS for D56 was not conducted in the AFR population due to a sample size of fewer than 200 cases; therefore, no locuszoom plot is available for D56 in AFR. rs11549407 (no LD estimation for this rare variant) MAF: 0.005% in NFE, 0 in SAS, 0.003% in AFR; rs33915217 MAF: 0.00008% in NFE, 0.41% in SAS, 0.004% in AFR; rs334: 0.004% in NFE, 0.089% in SAS, 6.26% in AFR. *P*-values are uncorrected and are from two-sided tests performed with approximate Firth logistic regression.

Of the meta-analysis significant associations, 126 were more significant in non-NFE ancestries (lead variant with the smallest *P* value) despite the much smaller sample size compared to NFE (Supplementary Fig. 6a): 83 with strongest signals in AFR, 37 in SAS, 5 in EAS and 1 in ASJ. Almost all 126 significant sentinel variants had MAF <0.5% in NFE; the median MAF enrichment compared with NFE is highest in AFR (MAF_AFR_/MAF_NFE_) = 828.49, followed by EAS and SAS with a relatively wide range of enrichment (Supplementary Fig. 6b). For example, we observed ancestry-specific associations in the *HBB* locus (Extended Data Fig. 3). The lead variant, rs334 (chr. 11:5227002:T:A), a missense variant in the *HBB* gene, is the primary cause of sickle cell disease, resulting in abnormal haemoglobin. Despite causing sickle cell disease, rs334-A is specifically common in AFR, driven by its protective effect against malaria and selective advantage in AFR^31^. One *HBB* splice site variant rs33915217 (chr. 11:5226925:C:G) is associated with β-thalassaemia and anaemia with elevated frequency specifically in SAS, potentially shaped by genetic drift, founder effect or unknown selective advantage^32^. Another *HBB* nonsense variant, rs11549407 (chr. 11:5226774:G:A), is associated with thalassaemia and anaemia detectable only in NFE given the large size (*P* value < 5.6 × 10^−62^, *β* = 6.9). rs11549407-A introduces a premature stop codon, leading to an unstable haemoglobin molecule, but it has not been shown to confer protection against malaria or other pathogens. Under the same selection pressure of malaria, a *G6PD* missense variant rs1050828 (chr. X:154536002:C:T), which causes the G6PD deficiency and haemolytic anaemia but provides protection against severe malaria, reaches high frequency in AFR (14.7%) but remains rare in NFE (0.005%). It is an AFR-specific GWS signal linked to increased reticulocyte and bilirubin levels, indicating compensatory release triggered by haemolysis.

### Loss-of-function variants in WGS

Naturally occurring human genetic variation known to result in disruption of protein-coding genes provides an in vivo model of human gene inactivation. Individuals with loss-of-function (LoF) variants, particularly those with homozygous genotypes, can therefore be considered a form of human ‘knockouts’. Studying human knockouts affords an opportunity to predict phenotypic consequences of pharmacological inhibition. Besides putative LoF (pLoF) variants that can be predicted on the basis of variant annotation, ClinVar^23^ also reported pathogenic or likely pathogenetic (P or LP, respectively) variants with clinical pathogenicity. Among the 490,000 UKB WGS participants (GraphTyper dataset), we found that there are 10,071 autosomal genes with at least 100 heterozygous carriers and 1,202 autosomal genes with at least 3 homozygous carriers. Among the 81 genes recommended by the American College of Medical Genetics and Genomics (ACMG)^33^ for clinical diagnostic reporting, we found 7,313 pLoF, P or LP variants carried by 51,107 individuals. Furthermore, there are 81 homozygous carriers of pLoF, P or LP variants found in 14 ACMG genes, of which 56 participants carry mutations in DNA repair pathway genes such as *MUTYH*, *PMS2* and *MSH6* (Supplementary Table 11). Among them, a subset are clinically actionable genotypes with a confirmed functional impact in the corresponding inheritance mode. Further validation, and confirmation with ACMG diagnostic criteria, is needed to determine which variants are clinically actionable.

**Fig. 3:**
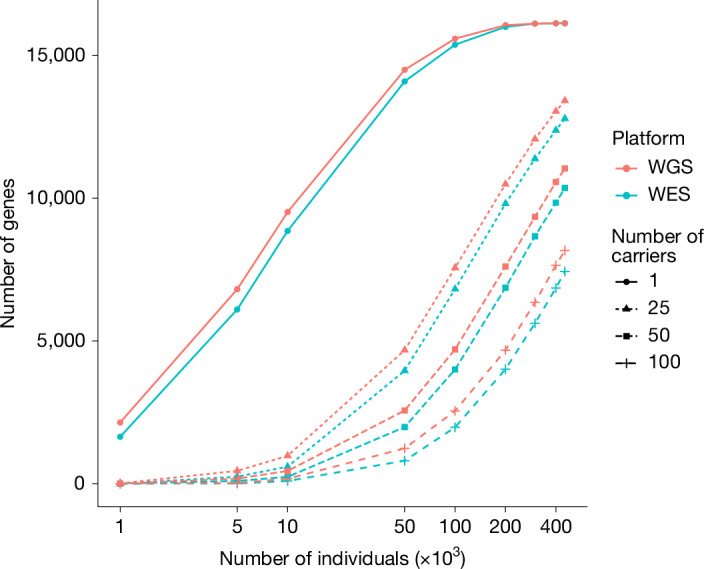
Observed number of genes in carriers of heterozygous pLoF, P or LP variants in WGS and WES. The number of autosomal genes (*y* axis) with at least 1, 25, 50 and 100 heterozygous carriers among the number of individuals (*x* axis) to the total number of 452,728 participants with both WES and WGS data.

Comparing the UKB WGS dataset versus the WES dataset, among the same set of 450,000 participants, about 16,000 autosomal genes harbouring pLoF, P or LP variants in ≥1 carriers in both WGS and WES. However, WGS enabled us to find more carriers of high-impact variants (for example, the median difference in the number of carriers is 44 more in the WGS dataset compared to the WES dataset for the gene sets with >100 carriers; Fig. 3). Partially attributable to quality control criteria (Supplementary Methods), this is also expected given the more even and deeper coverage in WGS.

### Rare-coding-variant association studies with WES and WGS

Gene-level collapsing analysis, in which aggregation of rare variants is tested for association with disease, has emerged as a powerful method for identifying gene–phenotype associations with high allelic heterogeneity^21,34^. So far, most collapsing analyses have used WES data^35^. We reasoned that the greater coverage of WGS compared to WES could increase power to detect gene–phenotype associations. We performed two collapsing analysis-based phenome-wide association studies (PheWAS) on an identical sample of 460,552 individuals using both WES- and WGS-based protein-coding regions (Supplementary Methods). All results for rare-variant collapsing analyses use the single-sample DRAGEN variant calls. In total, we tested for the association between 18,930 genes and 751 phenotypes (687 binary ‘first occurrence’ phenotypes and 64 quantitative traits that met our inclusion criteria; Supplementary Methods and Supplementary Table 12) using 10 non-synonymous and 1 synonymous control collapsing analysis models (Supplementary Table 13 and Supplementary Methods). We meta-analysed the separate ancestry strata and set the significance threshold at *P* value ≤ 1 × 10^−8^, which was previously empirically validated^21^.

**Extended Data Fig. 4:**
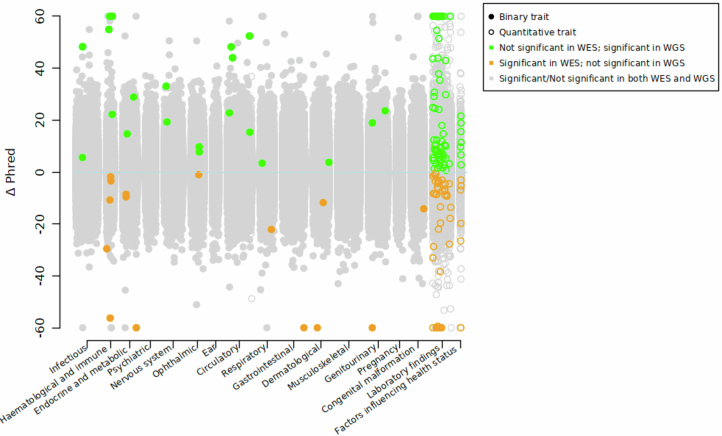
The change in Phred scores (−10*log10[p-values]) between the WGS and WES analyses for 12,963,003 binary genotype-phenotype associations (filled circle) and 1,167,322 quantitative associations (empty circle) stratified by chapter. For gene–phenotype associations that appear in multiple collapsing models, we display only those with the lowest P value within each dataset. The green circles indicate associations that were not significant in the WES analysis but were significant in the WGS analysis. The orange dots represent associations that were originally significant in the WES analysis but became not significant in the WGS analysis. The y axis is capped at ΔPhred = 60 (and −60), equivalent to a P value change of 0.000001.

In total, we identified 1,359 significant gene–phenotype associations, of which 87.4% (1,188) were significant in both the WES and WGS PheWASs (184 binary and 1,004 quantitative associations), 7.7% (105) were significant only in the WGS PheWAS (23 binary and 82 quantitative associations), and 4.9% (66) were significant only in the WES PheWAS (15 binary and 51 quantitative associations; Supplementary Table 14). There was high correlation between −log_10_[*P* values] derived from WES and WGS (Spearman’s rank correlation coefficient = 0.95, *P* < 2.2 × 10^−16^; Supplementary Fig. 7). Across both binary and quantitative traits, there were 29 genes with significant associations unique to WGS and 20 genes with significant associations unique to WES (Supplementary Fig. 8). Three genes uniquely associated with either technology are in the major histocompatibility complex region: *VWA7* (WES) and *HLA-C* and *C2* (WGS). Fewer than 3.3% of gene–phenotype pairs had an absolute difference in −10 × log_10_[*P* values] of greater than 5 units and fewer than 0.56% had greater than 10 units (Supplementary Fig. 9). Across all 14,130,325 gene–phenotype associations (significant and non-significant), there were 54,818 with greater than a 10-unit difference that achieved a lower *P* value in the WGS results, compared to 23,687 that achieved a lower *P* value in the WES results (Extended Data Fig. 4).

We identified 95 significant gene–phenotype associations with 15 genes recurrently mutated in clonal haematopoiesis and myeloid cancers as described previously^36^, which are potentially driven by somatic qualifying variants. Of these, 70 were detected by both technologies, 11 were unique to WGS and 14 were unique to WES. Associations unique to WGS included protein-truncating variants in *TET2* and other disorders of white blood cells (WGS *P* value = 3.62 × 10^−13^, OR = 8.08, 95% confidence interval (CI) = 5.02–12.40; WES *P* value = 4.23 × 10^−7^, OR = 6.18, 95% CI = 3.26–10.70). We also found an association between protein-truncating and predicted damaging missense variants in *SRSF2* and reticulocyte percentage (WGS *P* value = 1.92 × 10^−6^, *β* = 0.30, 95% CI = 0.17–0.42; WES *P* value = 3.7 × 10^−18^, *β* = 0.60, 95% CI = 0.47–0.74) significant only in the WES results (Supplementary Table 14).

Overall, although association results between the WES and WGS DRAGEN datasets are highly correlated, there are genes for which coverage is improved in WGS, resulting in modestly improved association statistics. One example is *PKHD1*, for which associations with three quantitative phenotypes were more significant in WGS than WES: γ-glutamyl transferase (WES *P* value = 4.63 × 10^−18^, *β* = 0.19, 95% CI = 0.15–0.24; WGS *P* value = 1.24 × 10^−19^, *β* = 0.20, 95% CI = 0.16–0.24), creatinine (WES *P* value = 3.85 × 10^−10^, *β* = −0.04, 95% CI = −0.06 to −0.03; WGS *P* value = 2.14 × 10^−12^, *β* = −0.05, 95% CI = −0.06 to −0.03) and cystatin C, which achieves significance only in the WGS data (WES *P* value = 3.02 × 10^−8^, *β* = −0.05, 95% CI = −0.07 to −0.03; WGS *P* value = 3.04 × 10^−9^, *β* = −0.04, 95% CI = −0.06 to −0.03; Supplementary Table 14). The number of samples with ≥10× coverage of *PKHD1* is lower in WES than WGS at specific protein-coding sites (Supplementary Fig. 10), demonstrating the value of WGS to ascertain variants and associations in regions not well captured by WES.

We calculated coverage statistics in the WES and WGS datasets for each protein-coding gene (Supplementary Table 15). There are only 638 genes in the WGS for which <95% of the protein-coding sequence had on average at least 10× coverage across the cohort, compared to around twice as many (1,299) in the WES dataset^21^. This improved coverage of some genes in the WGS data compared to WES demonstrates the value of WGS for improved discovery potential in some protein-coding regions.

### Rare-variant PheWAS of UTRs

To understand the contributions of rare UTR variants to phenotypes, we used the UKB single-sample DRAGEN WGS data to compile about 13.4 million rare (MAF < 0.1%) variants from both 5′ and 3′ UTRs of protein-coding genes across the 5 defined ancestries. We performed two multi-ancestry collapsing PheWASs: UTR alone and UTR plus protein coding.

**Fig. 4:**
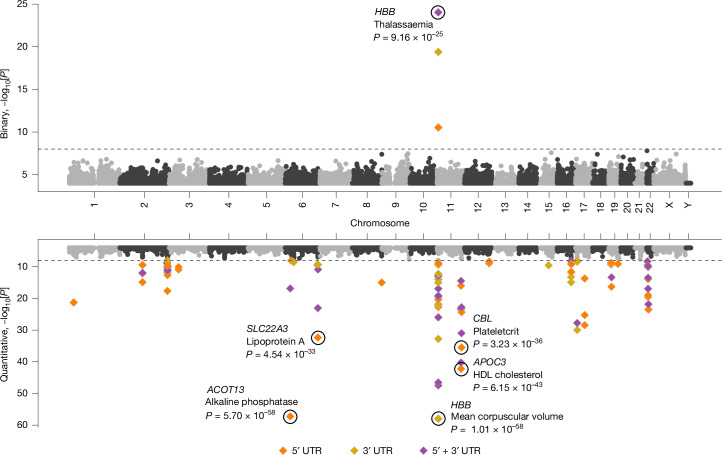
UTR-based collapsing analysis. Miami plot of UTR-based rare-variant PheWAS associations for 687 binary (top) and 64 quantitative (bottom) phenotypes across all 6 collapsing models. Significant 5′, 3′ and 5′ and 3′ combined associations are represented in different colours. The top significant binary associations and the significant quantitative associations with *P* value < 1 × 10^−30^ are labelled. *P* values are unadjusted and are from Fisher’s exact two-sided tests (for binary traits) and linear regression (for quantitative traits).

We tested the aggregate effect of UTR-alone qualifying variants on binary and quantitative phenotypes for 5′ UTRs alone, 3′ UTRs alone and 5′ and 3′ UTRs combined (Supplementary Table 12). Each was run using six collapsing analysis models to capture a range of MAF and CADD^37–39^ thresholds. Any UTR sites that overlapped a protein-coding site were omitted. Using a previously described *n*-of-1 permutation approach^21^, we confirmed that *P* value ≤ 1 × 10^−8^ is an appropriate significance threshold (Supplementary Methods). We observed 63 significant associations (1 binary trait and 62 quantitative traits) comprising 32 unique genes and 37 unique phenotypes (Fig. 4 and Supplementary Table 16). Many of these gene–phenotype associations have previously been identified with rare protein-coding variants or have GWAS support^38,39^. For example, 32 of 63 (51%) signals were also significant in the WGS protein-coding collapsing PheWAS already described, and 52 of 63 (83%) had a common variant within 500 kilobases (kb) significantly associated with the same phenotype in the UKB WGS Consortium GWAS already described (Supplementary Methods and Supplementary Table 16). The observed associations are likely to include some UTR variants that are causally linked to the phenotype, and some that are in partial linkage disequilibrium with nearby common variant associations.

We next explored the combined effect of rare UTR variants and protein-truncating variants using two different models. We observed 27 and 157 significant associations for binary and quantitative phenotypes, respectively (Supplementary Table 16). Ten associations that achieved significance in this UTR plus protein-coding PheWAS were not significant in the protein-coding-alone collapsing PheWAS, suggesting that those associations were augmented by incorporating UTRs (Supplementary Table 16). Furthermore, 27 suggestive (1 × 10^−8^ < *P* < 1 × 10^−6^) associations in the UTR plus protein-coding PheWASs did not reach this threshold in the protein-coding-alone collapsing PheWAS (Supplementary Table 16). For instance, *NWD1* is suggestively associated with kidney calculus (*P* value = 7.53 × 10^−7^, OR = 1.63) in the UTR plus protein-coding PheWAS, but not in the protein-coding-alone or the UTR-alone collapsing PheWASs. This is mostly driven by rare 3′ UTR variants (Supplementary Table 17), although the qualifying variants are distributed throughout the gene. No significant common variant associations were observed between *NWD1* (±500 kb) and kidney calculus in the UKB WGS Consortium GWAS; however, a common synonymous variant, rs773852, is associated with kidney calculus in a Chinese Han population^40^ Our study demonstrates the potential of WGS in identifying non-protein-coding variant to phenotype associations.

### Phenotypic effects of SVs

Associations identified in the previous UKB 150,119 release^22^ from the WGS consortium were mostly replicated. The new UKB release allows the identification of rarer SVs and assesses their impact on phenotypes. We present exemplary associations, anchoring on genes and variants that have a well-established association with phenotype.

Genes are typically affected by several SVs. Previously^22^, we highlighted an association of non-HDL cholesterol with a 14,154-bp deletion overlapping *PCSK9*, a gene encoding a proprotein convertase involved in the degradation of LDL receptors in the liver. In the current release, 13 SVs overlapping coding exons in *PCSK9* are found, carried by 163 individuals, bringing the total number of PCSK9 pLoF carriers to 1,124 The previously reported SV is the most common of the 13 variants, seen in 111 individuals. The carriers had (1.22 s.d.) lower levels of non-HDL cholesterol, with carriers of other *PCSK9* deletions collectively averaging 0.51 s.d. lower levels.

A 5,200-bp deletion on chr. 12: 56,451,164–56,456,364, is carried by 15 NFE individuals and it strongly associates with cataracts (OR = 25.3, *P* value = 6.3 × 10^−7^, MAF = 0.0015%). It deletes all 4 coding exons of *MIP* while preserving its 5′ UTR region and not affecting other genes. *MIP* encodes the major intrinsic protein of the lens fibre and rare deleterious missense, and LoF variants are linked to autosomal dominant cataract^41,42^.

The ACMG^43^ recommends reporting actionable genotypes in genes linked with diseases that are highly penetrant with established interventions. We previously reported^22^ that 4.1% of UKB individuals carry an actionable SNP or indel genotype. An additional 0.60% of individuals carry SVs predicted to cause LoF in autosomal dominant LoF, P or LP genes. If confirmed^44^, this increases the number of individuals with an actional genotype by 14.8%.

ClinVar^45^, a database of clinically significant variants, contains 2,256,088 records at present, but only 4,062 are SVs. Of these, 458 SVs presented here matched 486 (12.0%) in ClinVar. As expected, benign or likely benign variants have a higher frequency than P or LP variants (Supplementary Table 18). The large cohort and rich medical history allows us to assess the likely clinical impact of these variants and potentially refine the ClinVar classification.

Most ClinVar-annotated pathogenic SVs are very rare (MAF < 0.01%; Supplementary Table 18). One example is a 52-bp deletion on chr. 19: 12,943,750–12,943,802 in the first exon of *CALR* resulting in a stop gain. This recurrent somatic mutation^46–48^ is listed as pathogenic for primary myelofibrosis and thrombocythaemia is carried by 47 NFE individuals and 1 AFR individual. It strongly associates with measures of platelet distribution; most strongly with platelet width, effect 2.02 s.d. (95% CI = 1.72–2.34, *P* value = 3.1 × 10^−38^). It is present in the SNP and indel call set, but is not found in the WES data, despite being exonic.

Although most ClinVar variants are very rare in the UKB some have a higher frequency in the sub-cohorts. One example is a 2,502-bp deletion on chr. 2: 151,645,755–151,648,057 deleting exon 55 of *NEB*, linked with nemaline myopathy and traced to a single founder mutation^49^; it is carried by 33 individuals in the cohort, 17 of whom belong to the ASJ cohort. Another example is a 613-bp deletion on chr. 11 : 5,225,255–5,225,868 removing the first 3 exons of *HBB* seen in 19 individuals all belonging to the SAS cohort. The deletion has been annotated in ClinVar to be clinically significant for β-thalassaemia, and we find it to be associated with a 1.96 s.d. (95% CI = 1.49–2.43, *P* value = 5.4 × 10^−16^) decrease in haemoglobin concentration.

## Discussion

The UKB WGS project offers a groundbreaking opportunity to explore human genetic variation and its application to disease research. The vast dataset generated in this study will advance our understanding of human genetics and substantially impact drug discovery and development, disease risk assessment and precision medicine applications on a global scale. Furthermore, this work will provide essential insights into the contribution of rare non-coding variation to human biological variation and will facilitate the translation of human genetics into therapies over the next decade.

UKB WGS identified an 18.8-fold increase in variants compared with the imputed array and a greater than 40-fold increase compared with WES. This is consistent with multiple studies that highlight the power of WGS versus WES for identifying coding variants^5^, especially considering the decreased cost of WGS over time^6^. In accordance with previous efforts^14,22^, this information can also be used to identify regions that have a lower tolerance of variation. WGS allowed us to identify more genes harbouring pLoF, P or LP variants in more carriers, which offers more opportunities for evaluating gene targets in LoF heterozygous carriers or even human knockouts. WGS also allowed us to find many clinically relevant and disease-associated SVs.

Current human genomic reference and biobank data do not fully reflect the diversity of human populations and are still dominated by European ancestries^50^, thus limiting the detection of variation specific to non-European regions and leading to a fundamental bias in the understanding of the genetic basis of disease in diverse populations. In this study, we used cross-ancestry meta-analysis to confirm known associations and identify new ones with new indications and/or in non-European ancestries. Even though non-NFE ancestries had smaller sample sizes, 82 meta-GWS associations were found significant only in non-NFE ancestries (Supplementary Table 10), probably driven by selection pressure from regional-specific environment factors. For example, the missense causal variants of *HBB* and *G6PD* for sickle cell disease and anaemia, respectively, were >1,500× more common in AFR versus NFE, owing to their protection against severe malaria and the fact that 95% of malaria cases occur in Africa^51^. By contrast, a thalassaemia-causing *HBB* LoF mutation (rs11549407-A) and splice site variant (rs33915217-G) were most prevalent in NFE and SAS. These variants are rare in AFR and have no reported protective effects against malaria or other infectious diseases endemic to Africa. Whereas an *HBB* nonsense variant was detected in WES (allele frequency = 0.003%) but more enriched in WGS (allele frequency = 0.005%), the splice site variant was exclusively detected in WGS (not in WES or in imputed array genotypes), again highlighting the unique value of WGS.

To understand the impact of rare variants captured by WGS on human disease, we present a series of examples using collapsing analysis including protein-coding and non-protein-coding variants. Our observation that WGS can boost significance for certain genetic associations compared to WES in a collapsing analysis PheWAS context is consistent with other studies that show better coverage (and therefore better sensitivity to call variants) in WGS compared to WES for particular genes^52^. The benefit of WGS for protein-coding SNVs and indels is modest, which is expected and consistent with previous reports^53^. Defining qualifying variants in non-protein-coding regions remains challenging. In silico predictions of variant functional effect are less accurate in non-protein-coding regions than in protein-coding regions. Additionally, biological effects of variation in non-protein-coding regions are likely to be on average more modest than those in protein-coding regions. Nevertheless, our observation of significant rare-variant associations in UTRs, and a few phenotypes for which adding UTRs augments protein-coding signals, demonstrates the great potential of using this dataset to explore disease-relevant rare-variant associations in neglected non-protein-coding regions. Next steps could include further refining the non-protein-coding qualifying variant definitions with additional filters, conditional analysis to test for independence of non-protein-coding signals, expanding to other phenotypes, and expanding to other classes of non-protein-coding regions. In the UKB, additional data modalities provide a valuable opportunity to discriminate functionally important variants and therefore refine qualifying variant criteria. For example, a recent study using Olink plasma proteomics data in the UKB boosts signals by combining protein quantitative trait loci with protein-truncating variants in collapsing analyses^36^.

We have described and characterized this large WGS-based genetic study and provided examples showing that combining WGS data with the rich phenotypic data in the UKB gives new insights into the complex relationship between human variation and sequence variation. This resource not only can facilitate improved imputation performance for rare variants in individuals across five different ancestries^22,54^, but also will be useful for describing variation in complex regions, such as HLA, KIR and red blood cell antigen systems, and serve as a gold standard for future population-scale studies. We are confident that leveraging the combined expertise of scientists worldwide will lead to new insights that will meaningfully affect our understanding of human disease biology and thereby advance the search for safe and effective medicines.

### Reporting summary

Further information on research design is available in the Nature Portfolio Reporting Summary linked to this article.

## Online content

Any methods, additional references, Nature Portfolio reporting summaries, source data, extended data, supplementary information, acknowledgements, peer review information; details of author contributions and competing interests; and statements of data and code availability are available at 10.1038/s41586-025-09272-9.

## Supplementary information

Supplementary Information Consortia authorship, Supplementary Methods, Figs. 1–24, Tables 1–6, 8, 13, 17, 18 and 20–24, notes 1–4 and references.

Reporting Summary

Supplementary Table 7 GWAS_phenotypes_metadata.

Supplementary Table 9 Trans-ancestry meta-GWAS results for 68 quantitative traits (a) and 228 ICD-10 disease outcomes (b).

Supplementary Table 10 Associations with sentinel variants found significant only in non-NFE ancestries.

Supplementary Table 11 UKB WGS revealed heterozygous and homozygous carriers of pLoF, P or LP variants in the 81 ACMG genes.

Supplementary Table 12 Phenotypes included in region-based collapsing analysis PheWAS.

Supplementary Table 14 Significant (*P* ≤ 1 × 10^−8^) gene–phenotype associations identified in the coding PheWAS collapsing analysis across both WES and WGS datasets.

Supplementary Table 15 Gene informativeness.

Supplementary Table 16 Significant and suggestive gene–phenotype associations identified in the UTR PheWAS collapsing analysis across 5′ UTR, 3′ UTR, 5′ + 3′ UTR and coding sequence + 5′ + 3′ UTR.

Supplementary Table 19 GWAS Catalog GCST list.

## Data Availability

WGS data are available via the UKB research analysis platform (https://ukbiobank.dnanexus.com/landing), which is open to researchers from academic, charity, government and commercial organizations with an approved UKB project (https://www.ukbiobank.ac.uk/enable-your-research/apply-for-access). The Allele Frequency Browser is available at https://afb.ukbiobank.ac.uk/. Single-variant analysis results are available through the GWAS Catalog (study accession numbers are available in Supplementary Table 19). Rare-variant collapsing analysis association statistics are available through the AstraZeneca Centre for Genomics Research PheWAS Portal (http://azphewas.com/). SV association data are available at https://www.decode.com/summarydata/. All association summary statistics are made available for general research use and available at the time of access without access request. Human reference genome data GRCh38 are available at http://ftp.1000genomes.ebi.ac.uk/vol1/ftp/technical/reference/GRCh38_reference_genome/. Genome in a Bottle WGS samples are available at https://ftp-trace.ncbi.nlm.nih.gov/ReferenceSamples/giab/data/ and ENSEMBL annotation data at https://m.ensembl.org/info/data/mysql.html, versions 92 and 101.
