# Supplementary material for: Whole-genome sequencing of 490,640 UK Biobank participants
Source: Nature. 2025 Aug 6;645(8081):692–701. doi: 10.1038/s41586-025-09272-9 (PMC12443626; doi:10.1038/s41586-025-09272-9)
Supplement: Supplementary file 2 — Reporting Summary [file 41586_2025_9272_MOESM2_ESM.pdf]

nature portfolio

Slave Petrovski
Adrian Cortes
Bjarni Halldorsson
Shuwei Li
Corresponding author(s):

Last updated by author(s): May 21, 2024

Reporting Summary

Nature Portfolio wishes to improve the reproducibility of the work that we publish. This form provides structure for consistency and transparency in reporting. For further information on Nature Portfolio policies, see our Editorial Policies and the Editorial Policy Checklist.

Statistics

For all statistical analyses, confirm that the following items are present in the figure legend, table legend, main text, or Methods section.

n/a	Confirmed	
☐	☒	The exact sample size (*n*) for each experimental group/condition, given as a discrete number and unit of measurement
☐	☒	A statement on whether measurements were taken from distinct samples or whether the same sample was measured repeatedly
☐	☒	The statistical test(s) used AND whether they are one- or two-sided<br>*Only common tests should be described solely by name; describe more complex techniques in the Methods section.*
☐	☒	A description of all covariates tested
☐	☒	A description of any assumptions or corrections, such as tests of normality and adjustment for multiple comparisons
☐	☒	A full description of the statistical parameters including central tendency (e.g. means) or other basic estimates (e.g. regression coefficient) AND variation (e.g. standard deviation) or associated estimates of uncertainty (e.g. confidence intervals)
☐	☒	For null hypothesis testing, the test statistic (e.g. *F*, *t*, *r*) with confidence intervals, effect sizes, degrees of freedom and *P* value noted<br>*Give P values as exact values whenever suitable.*
☐	☒	For Bayesian analysis, information on the choice of priors and Markov chain Monte Carlo settings
☐	☒	For hierarchical and complex designs, identification of the appropriate level for tests and full reporting of outcomes
☐	☒	Estimates of effect sizes (e.g. Cohen's *d*, Pearson's *r*), indicating how they were calculated

Our web collection on statistics for biologists contains articles on many of the points above.

Software and code

Policy information about availability of computer code

Data collection

No software was used for data collection. Software used to generate the data from sequencing reads is described elsewhere in this manuscript.

Data analysis

tool	version
bambi	0.14.0
bamseqchecksum	v2.0.79
BamQC	v1.0.0
bcl2fastq	v2.20.0.422
biobambam	v2.0.79
bgzip	1.9
BOLT-LMM	v2.4.1
bwa mem	v0.7.17
CADD	v1.4
ClinVar	version 20231007
Dipcall	v0.1
DRAGEN	v3.7.8
Ensembl Build	38.92
FastQC	0.11.5
GATK	v4.0.12

gnomAD	v3.1
GraphTyper	v2.7.5
Manta	1.4.0
minimap2	v2.10
MTR	N/A
Picard	2.18.26
Plink2	v20240318
R	v3.6.0
REGENIE	v3.2.5
REVEL	N/A
RTG Tools	v3.8.4
Samblaster	v0.1.24
samtools	1.9
SnpEff	v4.3
svimmer	v0.1
tabix	v0.2.6
	vcftools |v4.2
VEP	release 101, hg38
VerifyBamID	v1.1.3

For manuscripts utilizing custom algorithms or software that are central to the research but not yet described in published literature, software must be made available to editors and reviewers. We strongly encourage code deposition in a community repository (e.g. GitHub). See the Nature Portfolio guidelines for submitting code & software for further information.

Data

Policy information about availability of data

All manuscripts must include a data availability statement. This statement should provide the following information, where applicable:

- Accession codes, unique identifiers, or web links for publicly available datasets
- A description of any restrictions on data availability
- For clinical datasets or third party data, please ensure that the statement adheres to our policy

WGS data is accessed via the UK Biobank research analysis platform (RAP; https://ukbiobank.dnanexus.com/landing), which is open to researchers from academic, charity, government and commercial organizations with an approved UKB projectn (https://www.ukbiobank.ac.uk/enable-your-research/apply-for-access). Allele frequency browser is available at https://afb.ukbiobank.ac.uk/. Single-variant analysis results are available through the GWAS Catalogue (study accession numbers available in Suppl. Table 19). Rare variant collapsing analysis association statistics are available through the AstraZeneca Centre for Genomics Research (CGR) PheWAS Portal (http://azphewas.com/). SV association data is available at https://www.decode.com/summarydata/. Summary statistics are made available for general research use and available at the time of access without access request. Human reference genome GRCh38, http://ftp.1000genomes.ebi.ac.uk/vol1/ftp/technical/reference/GRCh38_reference_genome/. GIAB WGS samples https://ftp-trace.ncbi.nlm.nih.gov/ReferenceSamples/giab/data/ ENSEMBL https://m.ensembl.org/info/data/mysql.html , versions 92 and 101.

Research involving human participants, their data, or biological material

Policy information about studies with human participants or human data. See also policy information about sex, gender (identity/presentation), and sexual orientation and race, ethnicity and racism.

Reporting on sex and gender	Biological sex was determined from the genetic data. Association analyses were performed with all individuals and sex status used as a covariate. No individual-level data is shown. No gender-based analyses were performed or data presented.
Reporting on race, ethnicity, or other socially relevant groupings	Genetic data was agregated across five ancestry groups defined with the genetic data. The analysis performed to derive such labels is described in Methods. Association analysis were performed per ancestry group and population stratification within group was controlled with principal components covariates.
Population characteristics	In association analysis we controlled for age and biological sex. Age was determined at baseline when individuals attended the first assessment in UK Boiobank. For controlling for population structure we used principal components as covariates derived from the genenotype information.
Recruitment	No recruitment was performed for this study. The recruitment in UK Biobank is described elsewhere and we reference such studies.
Ethics oversight	The UKB phenotype and genotype data were collected following an informed consent obtained from all participants. The North West Research Ethics Committee reviewed and approved UKB's scientific protocol and operational procedures (REC Reference Number: 06/MRE08/65). Data for this study was obtained and research conducted under the UKB applications license numbers 24898 and 68574.

Note that full information on the approval of the study protocol must also be provided in the manuscript.

Field-specific reporting

Please select the one below that is the best fit for your research. If you are not sure, read the appropriate sections before making your selection.

☒ Life sciences ☐ Behavioural & social sciences ☐ Ecological, evolutionary & environmental sciences

For a reference copy of the document with all sections, see nature.com/documents/nr-reporting-summary-flat.pdf

Life sciences study design

All studies must disclose on these points even when the disclosure is negative.

Sample size	Whole-genome sequencing was performed in all individuals where biological sample was available and this was of sufficient quality for the sequencing protocol.
Data exclusions	The sequencing of 914 participants failed due to either insufficient or poor-quality DNA, for a total of 490,640 successfully sequenced individuals. An additional 91 individuals withdrew consent from the time of start of sequencing until commencement of joint calling.
Replication	No replication was attempted for the findings presented here. The data resource presented here is first of its kind. Associations presented here are either known and validated elsewhere or illustrated as potential uses of this data resource.
Randomization	Participant's samples were received as provided by UK Biobank and sent for sequencing to two distinct centres. Samples were not ascertained by any clinical end-point, and the biobank was sequenced for all participants where possible.
Blinding	Blinding was not relevant to this study. There was no intervention that was not already randomised before recruitment of study participants.

Reporting for specific materials, systems and methods

We require information from authors about some types of materials, experimental systems and methods used in many studies. Here, indicate whether each material, system or method listed is relevant to your study. If you are not sure if a list item applies to your research, read the appropriate section before selecting a response.

Materials & experimental systems

n/a	Involved in the study
☒	☐ Antibodies
☒	☐ Eukaryotic cell lines
☒	☐ Palaeontology and archaeology
☒	☐ Animals and other organisms
☒	☐ Clinical data
☒	☐ Dual use research of concern
☒	☐ Plants

Methods

n/a	Involved in the study
☒	☐ ChIP-seq
☒	☐ Flow cytometry
☒	☐ MRI-based neuroimaging

Plants

Seed stocks	*Report on the source of all seed stocks or other plant material used. If applicable, state the seed stock centre and catalogue number. If plant specimens were collected from the field, describe the collection location, date and sampling procedures.*
Novel plant genotypes	*Describe the methods by which all novel plant genotypes were produced. This includes those generated by transgenic approaches, gene editing, chemical/radiation-based mutagenesis and hybridization. For transgenic lines, describe the transformation method, the number of independent lines analyzed and the generation upon which experiments were performed. For gene-edited lines, describe the editor used, the endogenous sequence targeted for editing, the targeting guide RNA sequence (if applicable) and how the editor was applied.*
Authentication	*Describe any authentication procedures for each seed stock used or novel genotype generated. Describe any experiments used to assess the effect of a mutation and, where applicable, how potential secondary effects (e.g. second site T-DNA insertions, mosiacism, off-target gene editing) were examined.*
